# Prevention of Post-Traumatic Stress Disorder After Trauma: Current Evidence and Future Directions

**DOI:** 10.1007/s11920-015-0655-0

**Published:** 2016-01-23

**Authors:** Wei Qi, Martin Gevonden, Arieh Shalev

**Affiliations:** Department of Psychiatry, New York University School of Medicine, 1 Park Ave, 8th Floor, 8-256, New York, USA

**Keywords:** Post-traumatic stress disorder, Prevention, Early treatment, Cognitive behavioral therapy, Pharmacotherapy, Targeted intervention

## Abstract

Post-traumatic stress disorder (PTSD) is a frequent, tenacious, and disabling consequence of traumatic events. The disorder’s identifiable onset and early symptoms provide opportunities for early detection and prevention. Empirical findings and theoretical models have outlined specific risk factors and pathogenic processes leading to PTSD. Controlled studies have shown that theory-driven preventive interventions, such as cognitive behavioral therapy (CBT), or stress hormone-targeted pharmacological interventions, are efficacious in selected samples of survivors. However, the effectiveness of early clinical interventions remains unknown, and results obtained in aggregates (large groups) overlook individual heterogeneity in PTSD pathogenesis. We review current evidence of PTSD prevention and outline the need to improve the disorder’s early detection and intervention in individual-specific paths to chronic PTSD.

## Introduction

The psychological effects of wars, disasters, terror, and other traumatic life events, can be deleterious and far-reaching. Post-traumatic stress disorder (PTSD) is the most widely researched consequence of traumatic events and as such epitomizes post-traumatic psychopathology. The clinical features comprising PTSD are event-related symptoms (intrusive recall of aspects of the event, avoidance of reminders, hyper-vigilance) along with dysphoria, hyperarousal, or anhedonia. PTSD is a prevalent consequence of both mundane traumatic events, such as road traffic accidents (7 to 26 %) [1] and protracted exposures to threat, such as wars (8 to 12.7 % among warzone-exposed US military personnel) [2].

PTSD may persist, unremitting, for years and decades in a subset of trauma-exposed survivors. The second wave of data collection (2013) of the nationally representative National Vietnam Veterans Readjustment Study (NVVRS, 1985), showed little improvement and frequent deteriorations of participants with PTSD [3]. Chronic PTSD is associated with poor physical health, inferior well-being, and unemployment [4]. The disorder is often comorbid with mood, anxiety, and substance use disorders [5, 6]. Co-occurring mental disorders worsen affected survivors’ outcome and increase the burden on public health.

Unlike other mental disorders, PTSD follows a distinct triggering event and has a clear onset point. Early PTSD symptoms develop within days of trauma exposure. Many trauma-exposed individuals are brought to the attention of emergency care services and helpers. These conditions create unique opportunities for detecting survivors at risk and providing preventive interventions. Conceptual models of PTSD’s pathogenesis, discussed below, have informed most early prevention techniques [7-9]. Despite these favorable attributes of PTSD, its systematic prevention is elusive at this point, and the disorder’s prevalence in the last four decades is remarkably stable, in both military personnel and civilians [10, 11].

The reasons that stagnate prevention of PTSD have not been fully elucidated, but several possibilities have been identified. Current preventive interventions were derived from evidence in chronic PTSD and may not properly engage the disorder’s pathogenesis. Efficient interventions have not been implemented on a large scale. Risk detection is imperfect. Service delivery is difficult when hostilities continue (e.g., during wars, mass relocation, protracted abuse). Studies have documented barriers to seeking help among symptomatic survivors. Community resources might not suffice for intense individual interventions.

Nonetheless, a rapidly growing body of work better informs our understanding of post-traumatic psychopathology, its neurobiological mechanisms, the resulting symptom trajectories, and putative trajectory moderators. This review outlines the better-researched theoretical models of PTSD and related interventions and discusses directions for future research and individual-specific prevention.

## Theoretical Models and Intervention Targets

### Interventions’ Taxonomy

Individuals’ reactions to traumatic events follow diverging trajectories. From quasi-universal disarray and distress, shortly after exposure, some survivors develop very few symptoms; others show transient and reversible initial symptoms, and a substantial minority keeps expressing severe non-remitting symptoms [12-14]. These findings define two primary goals for early interventions: Firstly, to mitigate the development of early symptoms, and secondly, to increase the likelihood of remission in those who develop symptoms (with special focus on the non-remitting subgroup). Interventions addressing the first goal include attempts to reduce the stressfulness of the traumatic event (e.g., ‘stress management,’ ‘need-based assistance’) [15, 16•], and interventions meant to reduce participants’ initial responses to the event or its encoding in memory. Studies addressing the second goal include specific intervention protocols delivered at different time intervals from the traumatic event to survivors identified as being at high risk for PTSD. The efficacy of the latter, therefore, hinges on proper risk detection at the early aftermath of trauma exposure. Individual risk prediction, however, is currently far from perfect.

### Promise and Current Limitations of Individual Risk Prediction

Empirically identified risk factors for PTSD are abundant. These can be temporally classified into pre-exposure ‘vulnerability’ factors, peri-traumatic factors and reactions directly related to the event, and post-exposure adversities. Pre-existing vulnerability factors range from neurobiological factors, such as genetic endowment and epigenetic regulation, through environmental factors, such as prior trauma exposure, family and personal psychiatric history, lower education, and stressful, resourceless living conditions, to behavioral factors, such as impaired executive function and higher emotional reactivity [17, 18]. Peri-traumatic factors include trauma intensity and type (e.g., intentional vs. unintentional), peri-traumatic symptoms, physiological arousal (e.g., heart rate) and gene expression. Post-exposure factors encompass social support (a protective factor), and ‘secondary’ stressors (e.g. unemployment as a result of the event) [19, 20].

Despite such an abundance of potential risk indicators, this knowledge has not yet been translated into individual risk prediction. One shortcoming of research to date is the use of statistical modeling that does not properly account for within-group heterogeneities. Studies universally use central tendency statistics, thereby implying that groups studied (e.g., rape victims, accident victims) are inherently homogeneous. However, trauma-exposed individuals are inherently heterogeneous, each bringing to the event his or her own array of vulnerability factors, environmental pressures (and provisions), psychological outfit and subjective appraisal of the traumatic event. Recent studies have used advanced analytic methods to define within - individual (as opposed to group average) symptom trajectories as the outcome of interest, and used machine-learning algorithms to make risk predictions. Several interchangeable sets of early risk indicators have been described including combinations of initial distress, early symptoms, injury severity, head injury, and subjective need for help [21, 22], allowing more versatile individual prediction. Current studies are exploring the clinical utility of such algorithmic solutions for calculating individual risk and predicting the need for intervention.

### Theory-Driven Interventions

**Fig. 1:**
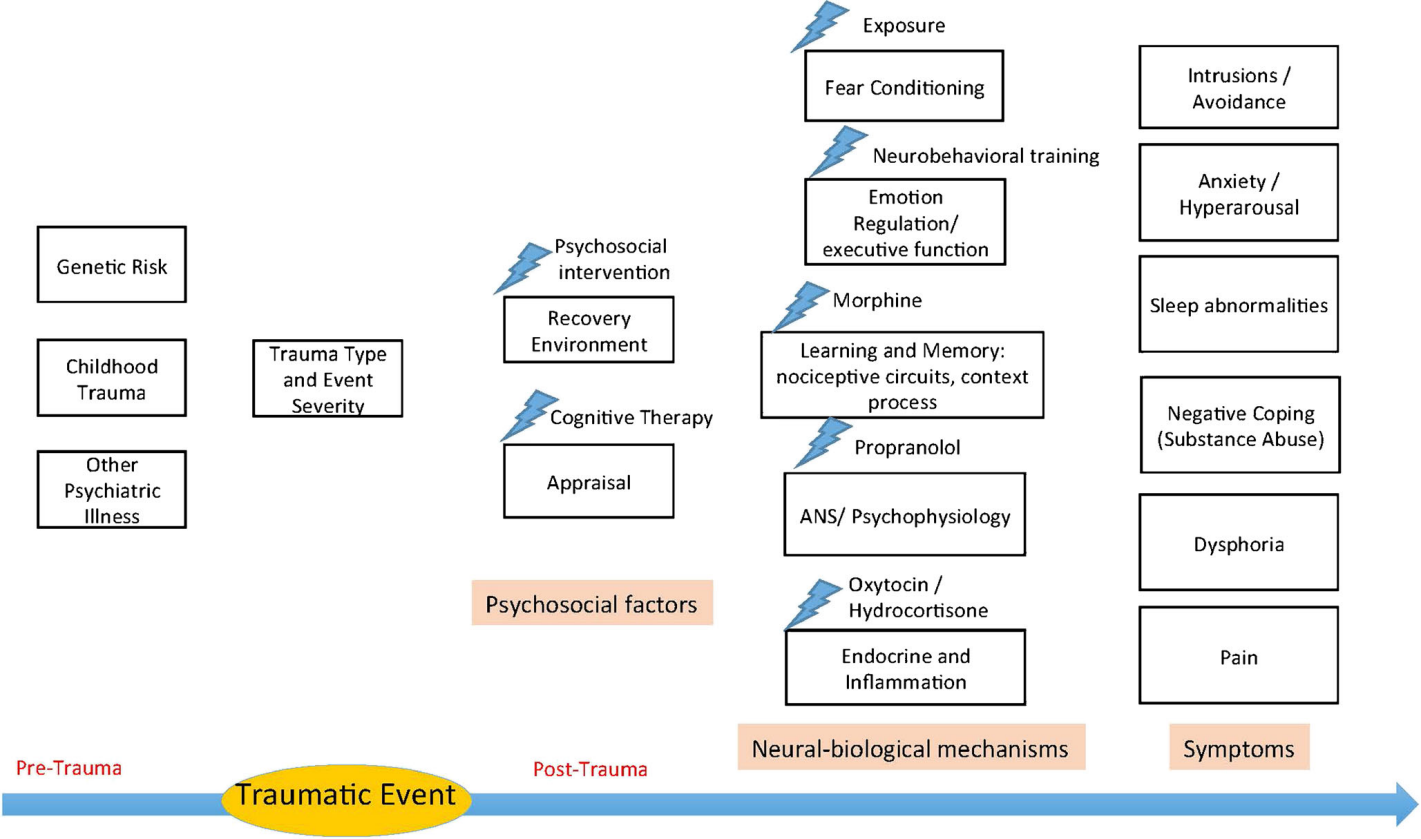
Prevention targets for post-traumatic psychopathology. The *bottom arrow* represents a timeline from pre-to post-trauma. Psychosocial factors and neural-biological mechanisms represent groups of potential targets for intervention.  indicates interventions targeting specific elements

Most preventive studies to date are theory-informed. Figure 1 presents the main theoretical models of PTSD pathogenesis, linking each model with specific interventions. The figure posits a progression from genetics and epigenetic vulnerability factors, childhood experience to peri-traumatic distress, and to specific pathogenic mechanisms operating during trauma exposure and its aftermath. The latter include psychological (appraisal of trauma, recovery environment) and putative neurobiological mechanisms underlying the pathogenesis of PTSD. The current review will only focus on secondary and tertiary prevention, which target the progression of psychopathology after the traumatic event. Interventions targeting elements in that progression (e.g., fear conditioning, emotion processing, initial neuroendocrine response) are associated with each element [23]. Considering such a progression shows that interventions’ timing and window of opportunity may be crucial for affecting relevant pathogenic mechanisms. For example, trauma memories may consolidate within hours of trauma, or during the first night sleep such that interventions designed to disrupt initial memory consolidation (pharmacological or psychological) must be provided within such timeframe [24, 25]. Other mechanisms in posttraumatic psychopathology, such as changes in memory, context processing, and nociceptive circuits may also occur within a currently unmapped time frame, calling for time-dependent intervention delivery [26].

## Overview of Preventive Interventions

### Psychological or Behavioral Interventions

#### The Demise of Psychological Debriefing

Psychological debriefing was a widely used method in the 1980s–1990s which aimed at preventing long-term post-traumatic symptoms by promoting quick emotional processing of traumatic events shortly after trauma exposure [27]. Debriefing was offered to survivors of a potentially traumatic event without prior diagnosis or evaluation, exposure being considered as good-enough risk indicator. The method typically involved a single session within hours or a few days after trauma exposure, either in a group or individual, and included general education about trauma exposure and its effect, sharing and validation of individuals’ experiences, and preparation for future encounters [28]. The method has face validity and is still well-known and, therefore, may be expected by lay people when confronted with traumatic events. However, well-conducted studies showed no evidence of beneficial effects and even suggested that debriefing may have a negative effect on recovery [29-31]. After a negative Cochrane review was first published in 1997, most treatment guidelines have been updated to recommend against providing single session psychological debriefing on a routine basis for adults after trauma [32, 33].

#### Cognitive Behavioral Therapy (CBT)

Trauma-focused CBT can involve different strategies with distinct aims. Exposure-based CBT, exemplified by the prolonged exposure (PE) protocol [34], aims to achieve and maintain fear extinction through repeated exposure to trauma-related stimuli in a safe context, thereby providing a sense of control over reactions and reducing avoidance. Cognitive-based CBT challenges the patient’s beliefs about the meaning and current implication of the trauma. It does so in order to change the way patients react to trauma-related reminders, to remove behavioral restrictions and rules derived from the traumatic experiences, and to reduce negative appraisal of self and others. CBT is offered individually or in a small group, to people who report symptoms (otherwise there are no ‘intervention targets’), and typically involves several weekly sessions, homework, and in vivo training exercises. The treatment may continue for over 3 months and requires significant skills from the therapists.

Trials of exposure-based CBT have generally demonstrated moderately positive results in reducing PTSD or other symptoms in the long term (Table 1). Rothbaum et al. (2012) conducted a study using modified Prolonged Exposure (PE) in rape, assault, and motor vehicle accidents survivors around 12 h after trauma, and found lower PTSD symptoms in the intervention group at 4 and 12 weeks after trauma, mainly for sexual assault victims. The same cohort also showed that PE might mitigate symptoms of PTSD in genetically predisposed individuals [35, 36•]. Bryant et al. (2008) found 5 weeks of exposure-based CBT to be effective in reducing PTSD in participants who met acute stress disorder diagnostic criteria [37]. Bisson et al. (2004) found a reduction of PTSD symptoms at 13 months—but not 3 months after the traumatic events [38], while a small study with 3 weeks of PE did not find significant symptom improvement in the PE group compared to supportive counseling [39].

**Table 1:** Psychological and behavioral Interventions for PTSD: Randomized Control Trials

Study	Design: groups (*n*)	Population	Intervention starting time*	Treatment sessions/ duration	Assessment time points*	Outcome measures	Results
Bryant et al. 1998 [50]	Exposure based CBT (12) SC (12)	MVA, industrial accidents survivors with ASD	<2 weeks	5 weekly 1.5-h sessions	<2 weeks, 2 and 6 months	CIDI, IES, BDI, STAI	Fewer PTSD cases, and intrusive, avoidance, and depressive symptoms in CBT group at follow-ups
Bryant et al. 1999 [51]	PE (14), PE + anxiety management (15) SC (16)	MVA, non- sexual assault survivors with ASD	<2 weeks	5 weekly 1.5-h sessions	<2 weeks, 2 and 6 months	CAPS-2; IES, BDI, STAI	Fewer PTSD cases in PE and PE + anxiety management groups at follow-ups
Ehlers et al. 2003 [52]	CT (28), self help (28), repeated assessment (29)	MVA Meeting PTSD diagnosis, PDS ≥ 20	Around 3 months, < 6 months	Up to 12 weekly sessions. 1^st^ session 90 min, afterwards 60 min	2, 3, 6, and 12 months	CAPS, PDS, BAI, BDI, disability report	Fewer PTSD cases, less PTSD, depression, anxiety symptoms, and reduced disability in CT group at follow-ups
Bisson et al. 2004 [38]	Exposure based CBT (76) Standard care (76)	Physical injury, meet PTSD criteria on PDS, HADS >15, or IES >35	5–10 weeks	4 weekly 1-h sessions	1–3 weeks, 12 weeks, and 13 months	CAPS, IES	Fewer PTSD symptoms, similar PTSD cases in CBT group at follow-ups
Bryant et al. 2006 [53]	Exposure based CBT (33) CBT + hypnosis (30) SC (24)	MVA, non-sexual assault survivors with ASD	<2 weeks	6 weekly sessions	Pre-treatment, Post-treatment, 6 months and 3 years	CAPS, IES, BDI, STAI	Fewer PTSD cases in CBT and CBT + hypnosis groups at follow-ups
Foa et al. 2006 [54]	Brief CBT (31) Assessment alone (30) SC (29)	Female victims of assaults, meet symptom criteria for PTSD	1 month	4 weekly 2-h sessions	Pre-treatment, post-treatment, 3 and 9 months	SCID, PSS, BDI, BAI, SAI	Greater decreases in PTSD severity in CBT group after treatment and at 3 months. No group difference at 9 months.
Sijbrandij et al. 2007 [40]	Brief CBT (79) Waitlist Control (64)	Assault, accidents, witness, etc. Meeting acute PTSD	<3 months	4 weekly 120-min sessions	Pre-treatment, 1 week and 4 months after treatment	SCID	Accelerated recovery in the CBT group after treatment; no difference in long term outcome
Bryant et al. 2008 [37]	Exposure based CBT (30),Cognitive restructuring (30) Waitlist (30).	MVA, non-sexual assault, preliminary diagnosis of ASD	<1 month	5 weekly 90-min sessions with daily homework	Pre-treatment, post-treatment, 6 months	CAPS, BDI, BAI, PTCI	Fewer PTSD in CBT group, not in cognitive restructuring group at follow-ups
Freyth et al. 2010 [39]	PE (19), SC (21)	Accident, assault victims, with ASD diagnosis	21 days	3 weekly sessions (90, 60, 60 min)	Pre-treatment, 1 week after treatment, 3 months, 4 years (telephone)	IES-R, DQ, PTCI, STAI, BDI	No significant group difference in symptomatic improvement.
Irvine et al. 2011 [43]	CBT (96) Usual care (97)	Cardioverter defibrillator patients	<2 months	8 weekly telephone-based counseling sessions	Before hospital discharge, 6 and 12 months	HADS, IES-R, Crown-Crisp Experiential Summary, SF-36	Significant PTSD symptom improvements in CBT group at follow-ups
Shalev et al. 2012 [41•]	PE (63), CT (40), Escitalopram (23), placebo (23) Waiting List/ Delayed PE (93)	ER trauma survivors, PTSD diagnosis (without duration criteria)	20 days	12 weeks	9 days, 20 days, 5 months, 9 months	CAPS, SCID, PSS-SR	Lower 5-month prevalence of PTSD in PE and CT. No 9-month difference between early and delayed PE
Rothbaum et al. 2012 [36•]	Modified PE (69) Assessment only (n = 68)	Rape, assault, MVA, meet Criterion A	12 hours	3 weekly 60-min sessions	12 h, 4 and 12 weeks	PSS-I, BDI, PDS	Lower PTSD symptoms at follow-ups, especially in sexual assault victims
Brunet et al. 2013 [55]	Dyadic CBT (37), control (37)	MVA, other accidents, physical assault, meet Criterion A1 and A2	About 25 days	2 sessions, 2 weeks apart	Pre-treatment (21 days), mid-treatment (35 days), post-treatment (3 months)	IES-R, CAPS, SCS, SAS-SR	Less PTSD symptoms in dyadic CBT group post-treatment
Shaw et al. 2014 [56]	TF-CBT 6 session (33), TF-CBT (29), comparison (1-session education) (43)	Mothers of pre-mature infants, above clinical threshold in any one of BAI, BDI, and SASRQ	2 weeks	6 or 9 sessions, 1–2 sessions/week, 45–55 min/session	1–2 weeks after childbirth, 4/5 weeks, 6/7 weeks (for 9-session TF-CBT group) and 6 months	DTS, SASRQ, BDI-II, BAI, MINI	Significantly fewer PTSD, anxiety and depression symptoms in CBT group at follow-ups. No difference in effect size between 6-and 9-session interventions.

*ASD* acute stress disorder, *BAI* beck anxiety inventory, *BDI* beck depression inventory, *CAPS* clinician administered PTSD scale, *CBT* cognitive behavioral therapy, *CIDI* composite international diagnostic interview, *CT* cognitive therapy, *DQ* dissociation questionnaire, *DTS* davidson trauma scale, *ER* emergency room, *HADS* hospital anxiety and depression scale, *IES* impact of event scale, *IES-R* impact of event scale – revised, *MINI* mini international neuropsychiatric interview, *MVA* motor vehicle accident, *PDS* posttraumatic diagnostic scale, *PE* prolonged exposure, *PSS*, PTSD symptom scale, *PSS-I* PTSD symptom scale – interview; *PSS-SR* PTSD symptom scale – self-report, *PTCI* post-traumatic cognitions inventory, *PTSD* post-traumatic stress disorder, *SAI* standardized assault interview, *SAS-SR* social adjustment scale – self-report, *SASRQ* stanford acute stress reaction questionnaire, *SC* supportive counseling, *SCID* structured clinical interview for DSM disorders, *SCS* social constraints scale, *SF-36* short form (36) Health Survey, *STAI* state-trait anxiety inventory, *TF-CBT* trauma-focused cognitive behavioral therapy. *Time indicators (e.g,. 4 week, 3 months) are based on time from traumatic events if not specified

CBT without in-session exposure has shown effectiveness in some but not all studies. Sijbrandij et al. (2007) compared CBT to waitlist control subjects with acute PTSD and found that CBT accelerated recovery, but makes no long-term difference [40]. Shalev et al. (2012), found that cognitive therapy fared as well as prolonged exposure 9 months [41•] after trauma exposure. However, neither intervention separated from non-intervention at 3 years [42]. Individuals classified as non-remitting [14] in that cohort were also treatment refractory.

Modifications of clinician-administered CBT, meant to make treatment more affordable or accessible, showed varied results. Irvine et al. (2011) conducted a telephone-based CBT in patients with an implantable cardioverter defibrillator installed and reported significant improvements in PTSD symptoms in the CBT group [43]. Mouthaan et al. (2013) developed a self-guided internet-based intervention (Trauma TIPS) based on CBT to prevent the onset of PTSD symptoms. The result did not support the efficacy of Trauma TIPS [44]. Two negative studies of five sessions of early telephone-based CBT have recently been submitted for publication (*O'Donnell* ML, *Shalev AY* personal communication).

CBT is currently the mainstay of early prevention of PTSD. Several considerations, however, make its systematic implementation a major challenge. First, CBT might not be needed in a large proportion of symptomatic survivors. A meta-analysis of early interventions has indicated that CBT is only efficient in participants with diagnosable PTSD at treatment onset [45, 46], and results from the Jerusalem Trauma Outreach and Prevention Study (J-TOPS) have similarly shown that survivors with sub-threshold PTSD symptoms equally recover with or without CBT [41•]. A study by Rothbaum et al. [36•] suggests that efficacy of early CBT is strongly dependent on the type of traumatic event. CBT was most effective for sexual assault victims, had a marginal effect among accident victims, and was not effective for victims of physical assault. Additionally, CBT was found to equally reduce chronic PTSD symptoms when delivered 1 or 5 months after the traumatic event [41•] and its initial effect was conserved for 3 years [42].

CBT is consequently best positioned as a clinical intervention for identified and ascertained acute PTSD cases. It is optimally provided at some distance from the traumatic event, during which time survivors with transient symptoms recover. Survivors who recover with early CBT seldom relapse spontaneously but could remain sensitive to subsequent exposure. Importantly, early CBT leaves numerous survivors unimproved (e.g., 20 % of those treated in the J-TOPS) and thus should be supplemented by ‘second-step’ interventions, which unfortunately are very poorly mapped, if at all. CBT is therefore a ‘must try’ in symptomatic trauma survivors, for many of whom it may shorten symptom duration by months and years.

### Pharmacological Interventions

Various pharmacological agents have been examined in the prevention of post-traumatic symptoms (Table 2). A Cochrane review in 2014 concluded that in general, there is moderate quality evidence for the efficacy of hydrocortisone, and no evidence for propranolol, escitalopram, temazepam, and gabapentin [47]. This field is rapidly developing as the neurobiological process underlying start to be clarified by more studies.

**Table 2:** Pharmacological Early Intervention for PTSD: Randomized Control Trials

Study	Design: groups (*n*)	Population	Intervention starting time*	Treatment duration	Assessment time points*	Outcome measures	Results
Schelling et al. 2001 [66]	Hydrocortisone 18 mg/kg/h iv (9), control (11)	Septic shock	During shock phase in hospital	6 days + 12 days taper	2+ years (31 months)	SCID traumatic memory PTSS-10	11 % PTSD in placebo group, 64 % PTSD in treatment group
Pitman et al. 2002 [60]	Propranolol 40 mg 4x/day (11); placebo (20)	ER trauma survivors, meet A1 + A2 criteria	<6 h	10 days + 9 days taper	1 month, 3 months	CAPS, psychophysiological imagery procedure	Lower mean of CAPS score, and less physiologic responders in propranolol group during follow-ups
Schelling et al. 2004 [67]	Hydrocortisone 10 mg/h iv (26); control (22)	High risk inflammation patients after cardiopulmonary bypass	Before surgery	24 h + 3 days taper	6 months	Traumatic memory PTSS-10	Slightly lower symptoms in treatment group at 6 months, weak evidence
Stein et al. 2007 [61]	Propranolol 40 mg 3x/day(17); gabapentin 400 mg 3x/day(14); placebo (17)	Physical injury in trauma center	<48 h	2 days uptitration, 8 days acute treatment, 4 days taper	In hospital, 1, 4, and 8 months	PCL-C, CES-D, ASDS, CIDI	Neither study drug showed a significant benefit over placebo on depressive or posttraumatic stress symptoms
Hoge et al 2012 [62•]	Propranolol 240 mg/day (22), placebo (21)	ER trauma survivors, meet A1 + A2 criteria	<12 h	10 days + 9 days taper	4 weeks, 12 weeks	CAPS, SCID	CAPS score and PTSD diagnosis no difference
Shalev et al. 2012 [41•]	PE (63), CT (40), Escitalopram 20 mg/day (23), placebo (23), waiting list/ delayed PE (93)	ER trauma survivors, PTSD diagnosis (without duration criteria)	20 days	12 weeks	9 days, 20 days, 5 months, 9 months	CAPS, SCID, PSS-SR	No difference between escitalopram and placebo groups
Delahanty et al. 2013 [48•]	Hydrocortisone 20 mg 2x/day. oral (31); placebo (33)	Traumatic injury, PDEQ > 27	<12 h	10 days + 6 days taper	ER, 1 month, 3 months	CAPS, CES-D, SF-36	Lower CAPS, more improvement in depression and quality of life in treatment group at follow-ups
Suliman et al. 2015 [68]	Escitalopram 20 mg/day (12); placebo (17)	Assault, MVA, witnessing, met intrusion or hyperarousal criteria of ASD	<4 weeks	24 weeks	<4 weeks, 1, 3, 6, 8, 10 months	CAPS, CGI, MINI, MADRS, VAS-D/A, SDS	No difference between groups

*ASD* acute stress disorder, *ASDS* acute stress disorder scale, *CAPS* clinician administered PTSD scale, *CES-D* center for epidemiologic studies depression scale, *CGI* clinical global impression scales, *CIDI* composite international diagnostic interview, *CT* cognitive therapy, *ER* emergency room,  *iv* intravenously, *MADRS* Montgomery Asberg Depression Rating Scale, *MINI* Mini International Neuropsychiatric Interview, *MVA* motor vehicle accident, *PCL-C* PTSD checklist – civilian version, *PDEQ* peritraumatic dissociative experience questionnaire, *PE* prolonged exposure, *PSS-SR* PTSD symptom scale – self-report; *PTSD* post-traumatic stress disorder, *PTSS-10* posttraumatic symptom scale, *SCID* structured clinical interview for DSM disorders, *SDS* Sheehan Disability Scale, *SF-36* Short Form (36) Health Survey, *VAS-D/A* Visual Analog Scale for Depression and Anxiety. *Time indicators (e.g., 4 weeks, 3 months) are based on time from traumatic events if not specified

#### Hydrocortisone

Hydrocortisone has been shown to be effective especially in patients who have never been treated for psychiatric disorders [48•]. A study recruited 64 trauma survivors in a level I trauma center and randomly assigned them to hydrocortisone and placebo group. At 3 months post trauma, no (0 %) hydrocortisone recipient and 3 (14 %) placebo recipients met full PTSD diagnostic criteria. PTSD symptom severity decreased over time in both groups, with hydrocortisone recipients reporting lower Clinician-Administered PTSD Scale (CAPS) scores than the placebo group (19.4 ± 4 vs.31.3 ± 3) [48•]. The underlying mechanism has not yet been established. One hypothesis is that hydrocortisone can facilitate extinction learning through both non-genomic and genomic effects [49]. Some also believe that high-dose exogenous hydrocortisone administered shortly after trauma may promote recovery through enhancing synaptic plasticity and connectivity. An animal model showed significantly increased dendritic growth and spine density, with increased levels of brain-derived neurotropic factor and decreased postsynaptic density protein 95 expression in steroid-treated stressed rats [57].

#### Propranolol

Propranolol is a beta-adrenergic antagonist that crosses the blood-brain barrier and, therefore, capable of reducing the central nervous system adrenergic drive associated with defensive threat responses. Experimental studies of propranolol in healthy subjects have shown that its administration prior to exposure to potentially traumatic narratives reduced the recollection of stressful elements of the narrative without affecting the general recall [58]. It was thereby positioned as a prime candidate to affect traumatic recall in PTSD. Early treatment with propranolol aims at preventing the over-consolidation of traumatic memories by blocking the memory-enhancing influence of stress hormones [59]. It therefore has to be started while memories of the trauma are still being formed and consolidated, preferably within hours of the traumatic event. An initial small-scale (*n* = 31) pilot study showed an efficacy of propranolol in reducing physiological responses to mental imagery of traumatic events 3 months after the event—but not on PTSD symptoms [60]. Two subsequent controlled studies [61, 62•], including one from the group that published the original study have also failed to show a preventive effect of propranolol. Because of its significant promise and strong theoretical basis, the documented gap between propranolol’s effect on physiological responses to trauma reminders (via mental imagery) and its lack of effect on PTSD symptoms might be interpreted as suggesting that the acquisition of traumatic memories in PTSD is not limited to amygdala-mediated threat conditioning and involves other modes of learning and memory [63].

#### Benzodiazepines

Benzodiazepines are gamma-amino butyric acid agonists and thereby enhance inhibitory transmission in many areas of the brain. They are used as tranquilizers and sleep inducers—also interfering with long-term potentiation and therefore with learning — and, in acute administration, with memory acquisition. They were positioned as capable of reducing excessive trauma-related learning, but then mostly tried for possible preventive effects on PTSD during the aftermath of traumatic events despite having no known effect on retrograde recall. In a prospective case-control study of 13 trauma survivors treated with the benzodiazepines clonazepam or alprazolam and 13 control cases matched by gender and symptom severity, benzodiazepine-treated patients were three times more likely to have PTSD at 6 months [64], a result which was replicated in another sample [65]. A ‘predator stress’ study in rodents similarly found that administering diazepam shortly after predator - odor exposure enhanced the acquisition of long-term fear responses (avoidance of open maze and exaggerated startle) [69]. While the exact mechanism of benzodiazepines’ PTSD-enhancing effect is unknown, it is possible that these compounds interfere with extinction learning, a critical phase in threat-response extinction, particularly when administered hours and days after trauma exposure. Remarkably, current evidence on benzodiazepines’ effect relies on small case series, whereas these compounds are widely used to mitigate acute response to stress. Further evidence is clearly needed, including the use of benzodiazepines to affect traumatic recall within minutes or hours from trauma exposure, that is, within the putative memory consolidation phase.

#### Morphine

Animal studies suggest that morphine can produce retrograde amnesia for contextual conditioned fear, possibly through decreasing cyclic adenosine monophosphate or activating N-methyl-D-aspartate receptors in the hippocampus [70]. Observational studies of hospital patients also suggested a possible beneficial effect of morphine administration within 48 h after trauma exposure to survivors who experience pain, reducing the likelihood of a PTSD outcome [71, 72]. A similar result was reported retrospectively in 696 military personnel with severe combat injury, in which not having PTSD was associated with higher likelihood of having received morphine—as per participants’ medical charts [73]. Given the retrospective nature of most studies, more research is needed to separate a specific effect of morphine from a generic ‘analgesic’ effect. Pain after trauma exposure is a potent predictor of PTSD. It is unclear, therefore, whether morphine has any preventive value in trauma survivors without physical pain.

### Other Approaches

Oxytocin is involved in emotion stress regulation, social engagement, and attachment. Olff et al. (2010) have suggested that oxytocin may buffer the development of PTSD by reducing fear responses and increasing social functioning [74]. The group is currently conducting a randomized control trial to examine the efficacy of intranasal oxytocin administration in preventing PTSD [75]. Neuropeptide Y (NPY) is another neuroendocrine candidate for intervention. An animal study showed that delivery of NPY to rats’ brains has a pronounced effect on reducing the development of PTSD-like symptoms, possibly through modifying stress-triggered dysregulation of hypothalamic–pituitary–adrenal axis and central noradrenergic activity [76]. Besides hormonal intervention, neural-behavioral trainings are also being developed to change negative emotional processing and enhance neurocognitive function. These trainings have shown positive results in treating anxiety disorders and depression [77-79]. With increasingly strong evidence of impaired emotion regulation and executive functions in PTSD, target-specific paradigms for early interventions will continue to emerge.

### Clinical Prevention in Context

Studies reviewed above summarize the ‘clinical’ implementation of preventive interventions; that is, the provision of specialized treatment to survivors screened or formally diagnosed within a medical care model. Challenging the restricted setting and medical model, Zatzick et al. (2004, 2013) evaluated a stepped collaborative care model, which introduced care managers to address patients’ unique needs using intervention modalities as required (e.g., components of CBT, Motivation Interview, pharmacotherapy). The care team repeatedly measured patient’s symptoms and adjusted levels of care accordingly. Results showed the feasibility and effectiveness of the method with reduced PTSD symptoms in the intervention group [16•, 80]. The need-based, multi-method model can be seen as an alternative to a single clinical intervention. It is likely a valuable component in a stepwise approach to early interventions, in which the ‘heavy artillery’ of full-fledged clinical interventions might be offered to those who fail to respond to earlier, cheaper, less demanding interventions in the need-based approach. By extension, it is generally true that current research has been limited to implementing single protocols and, as such, did not explore a sequential implementation of time-appropriate interventions. Such information should be sought and researched actively.

## Conclusions and Future Directions

The general picture emerging from this review is that the prevention of PTSD, despite its critical importance, is under-researched and inappropriately explored. Treatment protocols have been implemented regardless of sample heterogeneities and individual vulnerabilities [81], rudimentary theoretical assumptions have been hastily translated to haphazard case series, and randomized clinical trials fell short of informing the overall effectiveness of PTSD in a real world. This might be a typical situation in preliminary ‘proof of concept’ research, when treatment protocols must be rigidly implemented to ensure procedure reliability, and there is no empirical foundation to stratify samples—let alone modify treatment approaches according to needs and progress. In that sense, studies of the prevention of PTSD have successfully passed a first phase of searching in relative darkness.

Despite these critiques, there are many positive lessons to be learned from the current knowledge base. Taken seriously, they may and should guide better-informed efforts. Such efforts should concern the three tenets of efficient secondary prevention: risk assessment, understanding pathogenesis, and matching intervention techniques. Current research already points in that direction. While trauma-focused CBT received much support [45, 46, 82], it has been implemented without consideration of trauma survivors’ heterogeneity—the latter concerning variation in age, gender, trauma type, genetics and genomics, childhood experience, and recovery environment. Studies to date indicate that more refinement is needed—and possible. TF-CBT was reported to be more effective in victims of traffic accidents [83]; exposure therapy was shown to be more beneficial to sexual assault victims or people with high genetic risk [35, 36•]. Currently there is no evidence to support any treatment for trauma survivors already on a recovery trajectory. As Roberts et al. (2009) pointed out: current evidence does not support the routine implementation of any type of psychological intervention to all individuals after trauma [46].

From a risk assessment perspective, PTSD is likely multi-causal, and as such, individuals with differing vulnerabilities and different exposure and post-exposure circumstances may come to express the PTSD symptom complex through individual-specific pathways and be responsive to individual-specific interventions. One way to advance the prevention of PTSD is to better map the variety of paths leading to this condition and map those paths into subsets of trauma exposed individuals. Once such knowledge becomes available, personalized target-specific early interventions might replace generic treatment protocols, which in practice are effective for some but not for all.

Several steps should be taken in future studies. We need to step beyond diagnosis-based screenings and develop more complex accurate methods to predict individual risks of expressing debilitating symptoms and impairment after traumatic events. By enhancing prediction models, intervention studies can take the important step to select the most relevant sample for a more rigorous study design and the best clinical interest. At the same time, researchers need to continue exploring and confirming the underlying mechanisms of post-traumatic pathogenesis and, thereby, suggest new targets for novel interventions. These targeted intervention methods can allow clinicians to focus on both specific populations and specific pathological processes.
